# Aging with Meaning: What Motivates the Older Adults to End-of-life Care Volunteering

**DOI:** 10.1093/geroni/igaf122.896

**Published:** 2025-12-31

**Authors:** Asenath Lin, Vivian Lou, Gloria Chun, Felix Mak

**Affiliations:** The University of Hong Kong, Hong Kong, China (People’s Republic); The University of Hong Kong, Hong Kong, China (People’s Republic); The University of Hong Kong, Hong Kong, China (People’s Republic); The University of Hong Kong, Hong Kong, China (People’s Republic)

## Abstract

**Objective:**

This study examines the motivations and impacts of end-of-life care (EoLC) volunteering among older adults (aged ≥60), addressing critical gaps in aging societies where cultural taboos around death often limit engagement.

**Method:**

Under The Jockey Club End-of-life Community Care Project, a mixed-methods approach was employed, combining quantitative data from 212 participants and qualitative interviews with 11 volunteers.

**Findings:**

Using the Volunteer Functional Inventory (VFI)—a framework identifying six motivations (value, understanding, enhancement, protective, career and social). A Latent Profile Analysis revealed three distinct motivational profiles: a “Uniformly Low” group (40.5%) with minimal motivation across all factors, a “Uniformly High” group (41.8%) demonstrating strong engagement in all domains, and a “Protective-Career Oriented” group (17.7%) primarily driven by self-protective and career-related motives. Qualitative data revealed transformative impacts: volunteers gained EoLC knowledge, reported heightened meaning, reduced death fear, and contributed to breaking cultural taboos in Chinese communities. The “Uniformly High” profile aligns with qualitative intrinsic rewards (e.g., meaning, happiness), whereas “Protective-Career Oriented” underscores the fulfilment of personal needs in sustaining participation. Notably, participants motivated by altruism demonstrated sustained engagement and resilience in navigating service-related challenges. Conversely, those driven by protective motivations (e.g., alleviating negative emotions) were more susceptible to burnout overtime.

**Conclusion:**

Despite minimal quantitative shifts, findings highlight EoLC volunteering’s societal value in normalizing death discussions and its significance for older adults. Practical implications include tailoring recruitment and training to motivational profiles and leveraging volunteers as cultural change agents.

